# *Pseudomonas aeruginosa* transcriptome adaptations from colonization to biofilm infection of skin wounds

**DOI:** 10.1038/s41598-021-00073-4

**Published:** 2021-10-19

**Authors:** Peter D’Arpa, S. L. Rajasekhar Karna, Tsute Chen, Kai P. Leung

**Affiliations:** 1 Combat Wound Repair Group and Tissue Regeneration Department, US Army Institute of Surgical Research, JBSA Fort Sam Houston, San Antonio, TX USA; 2 The Geneva Foundation, Tacoma, USA; 3 The Forsyth Institute, Cambridge, MA USA

**Keywords:** Microbiology, Bacteria, Biofilms, Microbial genetics, Pathogens, Infectious diseases, Bacterial infection, Trauma

## Abstract

In burn patients *Pseudomonas aeruginosa* infection is a major cause of morbidity. Analysis of the pathogen’s gene expression as it transitions from colonization to acute and then biofilm wound infection may provide strategies for infection control. Toward this goal, we seeded log-phase *P. aeruginosa* (PAO1) into 3-day-old, full-thickness excision wounds (rabbit ear) and harvested the bacteria during colonization (Hrs 2 and 6), acute infection (Hr 24), and biofilm infection (Days 5 and 9) for transcriptome analysis (RNA-Seq). After 2–6 h in the wound, genes for metabolism and cell replication were down-regulated while wound-adaptation genes were up-regulated (vs. expression in log-phase culture). As the infection progressed from acute to biofilm infection, more genes became up-regulated than down-regulated, but the down-regulated genes enriched in more pathways, likely because the genes and pathways that bacteria already colonizing wounds up-regulate to establish biofilm infection are less known. Across the stages of infection, carbon-utilization pathways shifted. During acute infection, itaconate produced by myeloid cells appears to have been a carbon source because myeloid cell infiltration and the expression of the host gene, *ACOD1*, for itaconate production peaked coincidently with the expression of the PAO1 genes for itaconate transport and catabolism. Additionally, branched-chain amino acids are suggested to be a carbon source in acute infection and in biofilm infection. In biofilm infection, fatty acid degradation was also up-regulated. These carbon sources feed into the glyoxylate cycle that was coincidently up-regulated, suggesting it provided the precursors for *P. aeruginosa* to synthesize macromolecules in establishing wound infection.

## Introduction

*Pseudomonas aeruginosa* nosocomial spread presumably occurs from contaminated water or soil to people and from person to person via contaminated hands, equipment, or surfaces. In 2017 in the US, 32,600 hospitalized patients had *P. aeruginosa* infections and 2700 died. Patients at greatest risk for *P. aeruginosa* infection are the immunocompromised and those with invasive devices (e.g., ventilators) or surgical or burn wounds^1^. *P. aeruginosa* infection of burn wounds is associated with a significant increase in mortality^2^.

*P. aeruginosa* is intrinsically resistant to many antimicrobials and can easily develop resistance. New forms of resistance are particularly alarming, especially those that can spread via mobile genetic elements^1^. Some multidrug-resistant (MDR) *P. aeruginosa* are resistant to nearly all antibiotics, and 2–3% of carbapenem-resistant *P. aeruginosa* carry a mobile genetic element encoding carbapenemase^1^. Because people are dying from infections due to ineffective antibiotics, a “post-antibiotic” era is the reality^1^.

Nonetheless, antibiotic development has slowed. And antibiotics that are developed will have a finite lifetime of usefulness before resistance develops. Therefore, relying on traditional antibiotics alone to treat infections is not enough, alternative agents or targeting strategies are needed^1^. Targeting virulence factors is one alternative. Virulence factors enhance the fitness of pathogens at colonization/infection sites and targeting them can improve the immune control of pathogens and reduce pathogenicity. Although the effectiveness of virulence-factor targeting may be niche-specific, anti-virulence agents should save lives when applied to drug-resistant infections and could help preserve antibiotics’ effectiveness^1^. Virulence-targeting agents can synergize with antibiotics; for example, a disruptor of *P. aeruginosa* biofilm (glycopeptide dendrimers)^3^ and the immunomodulator and antimicrobial metabolite itaconate^4^ each increased sensitivity of *P. aeruginosa* to tobramycin, suggesting the usefulness of combination therapies^5^. Currently, only a few anti-virulence therapeutics are in clinical development^6^.

To understand the physiology during infection and to inform anti-virulence strategies, several previous studies have analyzed *P. aeruginosa* genome-wide gene expression under various conditions in vitro^7–9^ and in vivo^10–13^, including burn wound infection^14^. *P. aeruginosa* (strain PAO1) gene expression during wound infection has also been compared with gene fitness (transposon insertion sequencing, Tn-Seq), finding that the essentiality of genes generally did not correlate with their changes in expression from minimal medium to wound infection, except for metabolic genes^15^. The expression of core metabolic genes has also been associated with virulence across *P. aeruginosa* strains^16^. Furthermore,  the glyoxylate shunt impinges on virulence factor production^17–20^.

Here we have analyzed the *P. aeruginosa* transcriptome after log-phase bacteria were seeded into wounds and first colonized and then developed acute infection and finally biofilm infection. We previously reported the characterization of the wounds and the transcriptomic response of the wound tissue to this infection^21^.

## Results and discussion

**Figure 1:**
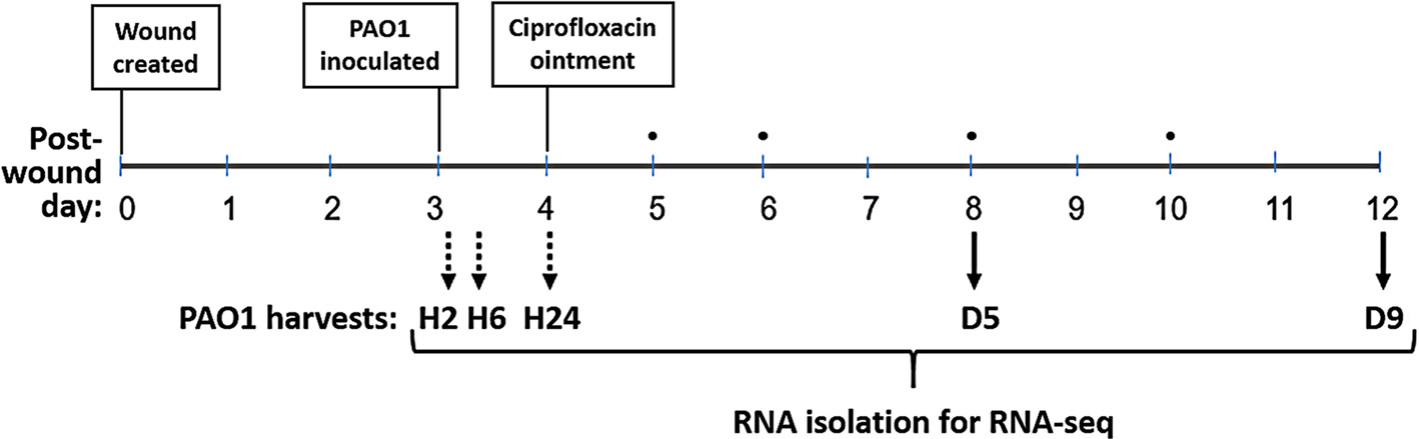
Study design*.* PAO1 from log-phase culture was pipetted (10^6^ CFU) into full-thickness excision wounds (rabbit ear) on post-wounding day 3. The bacteria were harvested at post-inoculation Hours 2, 6, and 24 (dotted black arrows). Other wounds infected for 24 h were treated for 24 h with topical ciprofloxacin ointment (Ciloxan), and then dressings were changed every day or two (TELFA AMD, black dots) until the bacteria were harvested on post-inoculation Days 5 and 9 (solid black arrows).

*P. aeruginosa* strain PAO1 from log-phase culture (~ 0.1 OD_600_) was seeded into wounds and then harvested for RNA-Seq after 2 and 6 h (H2 and H6, colonization), 24 h (H24, acute infection), 5 days (D5, less-mature biofilm infection) and 9 days (D9, more-mature biofilm infection) as depicted in Fig. 1. Our previous report describes the transcriptomic response of the rabbit ear wound tissue to this infection as well as the *P. aeruginosa* viable counts and morphology at each post-infection timepoint, including biofilm morphology characterized as aggregates on Days 5 and 9 that was confirmed by gene expression^21^.

### PAO1 global gene expression at post-wound inoculation times

**Figure 2:**
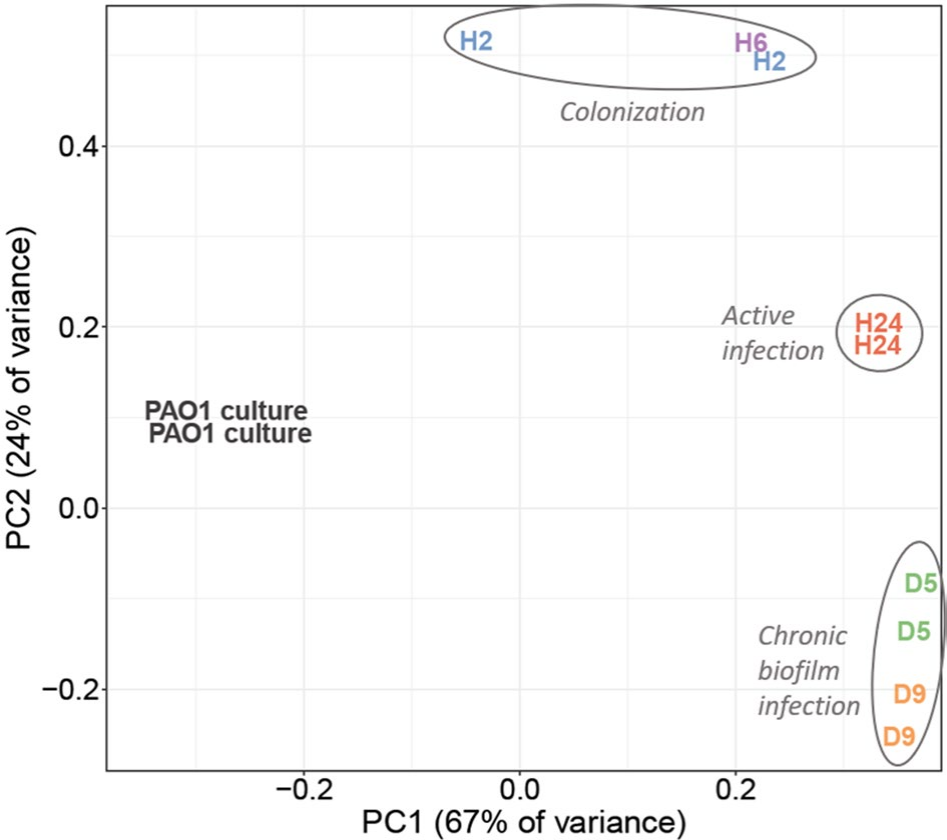
Principal component analysis of transcript counts of PAO1 in log-phase culture and at in-wound times. H2, Hour 2; H6, Hour 6; H24, Hour 24; D5, Day 5; and D9, Day 9.

We used principal component analysis to compare the transcriptomes of PAO1 in log-phase culture and as  wound infection developed. As shown in Fig. 2, the log-phase-culture transcriptomes are separated from the in-wound transcriptomes, which are further separated by infection stage: colonization (H2 and H6), acute infection (H24), and biofilm infection (D5 and D9).

Compared to expression in culture, slightly more PAO1 genes were up-regulated than down-regulated in wounds at all five post-inoculation times. As compared to the inoculum culture, in colonized wounds (H2 and H6 averaged), 30% of genes changed expression in either direction (FDR 0.05), whereas 56% of genes changed expression in either direction in biofilm infection (D5 and D9 averaged) (Supplementary Fig. S1, Supplementary File S1).

### PAO1 gene expression by genome position as wound infection developed

Gene expression viewed from the genes’ ordering across the genome shows localized gene expression reflecting operons as well coordinated gene expression over larger sectors. From this view, gene expression looks largely similar from Hour 2 to Day 9 of infection (Supplementary Fig. S2). Indeed, the direction of change of gene expression by Hr 2 (vs. culture) remained unchanged for half of all genes at every time point out to Day 9.

However, against this background, many genes’ expression changed significantly, most noticeably when comparing acute colonization (H2&H6) and biofilm infection (D5&D9). One outstanding change was in the 60 genes between PA2134 to PA2193 that began being overexpressed at 24 h post-infection (Supplementary Fig. S2). These gene’s coordinated expression was hypothesized in a previous report to be specific for *P. aeruginosa* infecting burns as compared to infecting lettuce ribs, mouse tumor, suspension culture or biofilm culture^14^. Our data show these genes change expression significantly from suspension culture only after being in the wound 24 h, possibly coincident with early biofilm formation. Several of these genes are involved or implicated in carbohydrate and polysaccharide biosynthesis and/or degradation, or the response to stress (see the gene annotations in Supplementary File S2)^14,22–24^. Genes in this region are regulated by the *rhl* quorum sensing system^25^, controlled by AlgU, RpoN and KinB^26^, up-regulated when PAO1 is exposed to human respiratory epithelia^27^ or oxidative stress, and may be involved in resistance to osmotic stress^28^.

### Changes in clusters of orthologous groups (COG) expression as wound infection developed

As the liquid culture inoculum developed from colonization to acute and then biofilm wound infection, the percentage of up- and down-regulated genes within clusters of orthologous groups of proteins (COG)^15,29,30^ confirmed the principal component analysis: the two colonization timepoints (H2 and H6) showed similar COG gene expression and the two biofilm-infection timepoints (D5 and D9) showed similar COG gene expression, while the acute-infection timepoint (H24) appeared intermediate between them (Supplementary Fig. S3).

The COG category most overrepresented with up-regulated genes at every timepoint was ‘Inorganic ion transport and metabolism’. This category’s up-regulation was previously reported for PAO1 infecting mouse burn and excision wounds after 40 and 96 h of infection, respectively^15^. Within this category, the genes in the subcategory ‘Iron acquisition and uptake mechanisms’ (99 total) were nearly all up-regulated at every in-wound timepoint vs. log-phase culture (Supplementary Fig. S4). Among these genes, the genes in the sub-category ‘Haem uptake—Has pathway’ (7 genes) on average became > threefold more up-regulated as the infection advanced from colonization to biofilm. These data further support the bacterial need for iron to establish and maintain wound infection.

**Figure 3:**
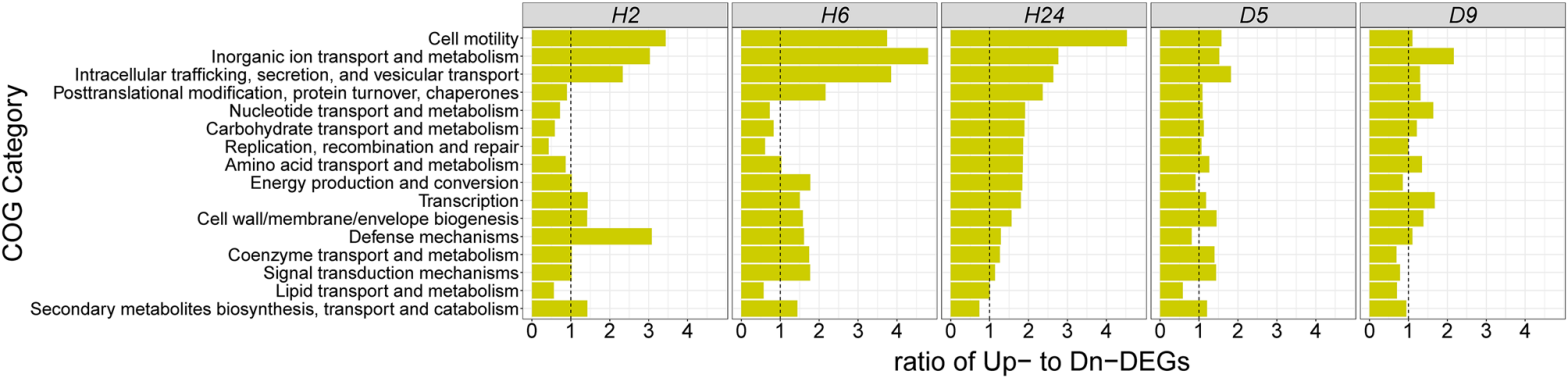
Ratio of up- to down-regulated PAO1 genes in COG categories at different times of wound infection vs. in culture. The COG categories are arranged from top to bottom by the highest to the lowest ratio of significantly up- to down-regulated genes at hour 24 (H24).

Next, we computed the ratio of significantly up- to down-regulated genes for each COG (Fig. 3). After PAO1 had inhabited the wounds for 2–6 h, genes in COGs related to elevated metabolism and cell replication—i.e., ‘Nucleotide transport and metabolism’, ‘Carbohydrate transport and metabolism’, ‘Lipid transport and metabolism’, and ‘Replication, recombination and repair’—were mostly down-regulated. Conversely at this earliest time point, the COGs with mostly up-regulated genes included ‘Defense mechanisms’, ‘Inorganic ion transport and metabolism’, ‘Intracellular trafficking, secretion, and vesicular transport’, and ‘Cell motility’, reflecting early adaptation to the wound environment.

After residing in the wounds for 24 h, the PAO1 inoculum increased in viable counts by 2 logs^21^. Coincidently, the COGs related to elevated metabolism and cell replication that were down-regulated at 2 h became mostly up-regulated after 24 h in the wound (Fig. 3), consistent with acute infection and proliferation. At this 24 h point, neutrophil and M1 macrophage infiltration and exudate was greatest^21^.

### Comparisons between stages of wound infection: gene expression by genome position

We computed differential gene expression between wound samples that were pooled (D5&D9:H2&H6) based on the above COG and the principal components analyses that show the two colonization time points (H2 and H6) have similar gene expression and the two biofilm infection time points (D5 and D9) have similar gene expression. Using the pooled samples (D5&D9:H2&H6), 21% of the PAO1 protein-coding genome (1203 genes) was differentially expressed (fold-change > 2 and FDR = 0.05) between colonization and biofilm infection (Supplementary File S3). We also computed differential expression between colonization and acute infection (H24:H2&H6), revealing differential expression of 16% of the genome (Supplementary File S4). Finally, between earlier and later biofilm infections, the fewest number of PAO1 genes was differentially expressed (D9:D5, Supplementary File S5); nonetheless, the plot of gene expression in *genomic order* between these earlier and later biofilm infections reveals numerous genomic regions of gene co-regulation (operons and longer regions) (Supplementary Fig. S5).

### Colonization vs. biofilm infection (D5&D9:H2&H6): COG expression changes

**Figure 4:**
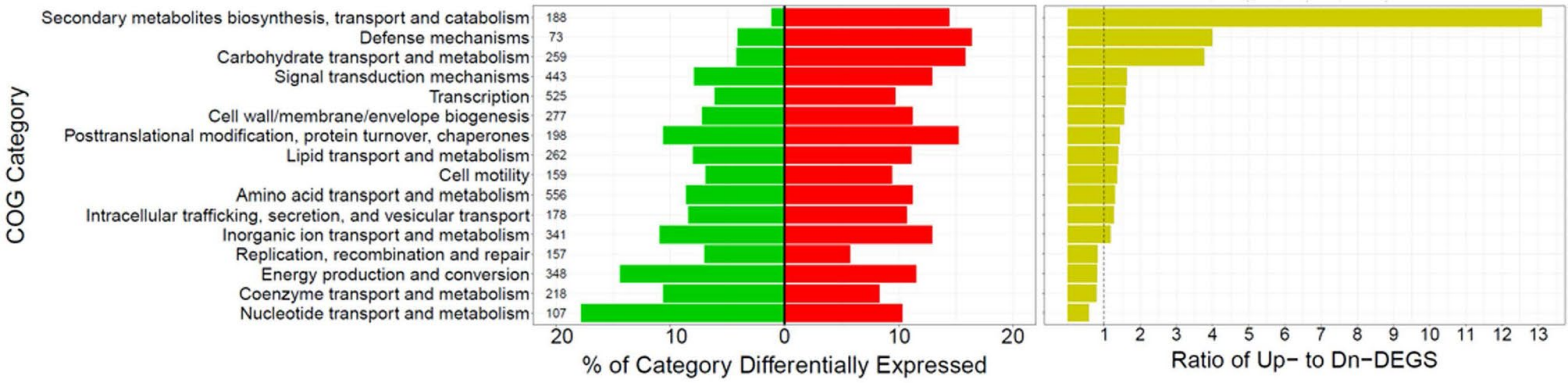
COG categories differentially expressed between biofilm and acute wound infection (D5&D9:H2&H6). The percent down-regulated (green) and up-regulated (red) genes in each COG category are graphed (right panel). The COG categories are ordered by descending ratio of up- to down-regulated genes (yellow, left panel). The numbers in the left panel are the counts of genes per COG category (*Pseudomonas Genome DB*, PAO1 COG mappings).

In biofilm infection vs. colonization, the COGs with the greatest ratio of up- to down-regulated genes were ‘Secondary metabolites biosynthesis, transport and catabolism’, ‘Defense mechanisms’, and ‘Carbohydrate transport and metabolism’ (Fig. 4). Conversely, COGs with mostly down-regulated genes during biofilm infection were ‘Replication, recombination and repair’, ‘Energy production and conversion’, ‘Coenzyme transport and metabolism’, and ‘Nucleotide transport and metabolism'.

### Colonization vs. biofilm infection (D5&D9:H2&H6): Pathway changes

We computed the over-representation of differentially expressed genes (> twofold, padj < 0.05) into pathways to better understand how *P. aeruginosa* adapts from colonization to biofilm wound infection. Although more PAO1 genes significantly increased (57%) than decreased (43%) expression from colonization to biofilm infection, the genes whose expression decreased in biofilm infection were over-represented in ~ threefold more pathways than the genes up-regulated in biofilm infection (Supplementary File S6), as summarized in Table 1.

**Table 1:** Pathways up- and down-regulated from acute (H2&H6) to biofilm infection (D5&D9).

Pathways^a^ with up-regulated genes overrepresented	p value**
Alginate biosynthesis (PseudoCyc, PseudoCAP, UniPathway)	8.74E−11, 1.37E−09, 1.83E−03
Starch and sucrose metabolism (KEGG, KEGG-InterPro, PseudoCAP)	2.13E−07, 7.46E−05, 1.01E−03
Apr type I secretion system (PseudoCAP)	2.73E−05
Pyrroloquinoline quinone biosynthesis (PseudoCAP, UniPathway)	1.47E−04, 4.63E−04
Ethanol oxidation pathway (PseudoCAP)	2.01E−04
l-2-Amino-4-methoxy-trans-3-butenoic acid (AMB) biosynthesis (PseudoCAP)	1.01E−03
Fructose and mannose metabolism (KEGG)	5.37E−03
Pyruvate decarboxylation to acetyl CoA (PseudoCyc)	6.67E−03
Anaerobic respiration (PseudoCyc)	4.40E−02

Pathways with down-regulated genes overrepresented	p value
Ribosome (KEGG)	9.36E−45
Adenosine ribonucleotides de novo biosynthesis (PseudoCyc)	1.11E−12
Sulfur metabolism (KEGG)	7.86E−09
Oxidative phosphorylation (KEGG, PseudoCAP)	2.55E−05, 4.704E−04
Type III secretion (PseudoCAP)	3.36E−05
Metabolic pathways (KEGG)	9.24E−05
Lipopolysaccharide biosynthesis (UniPathway)	1.89E−04
Citrate cycle (TCA cycle) (KEGG, PseudoCAP, KEGG-InterPro, UniPathway)	2.25E−04, 3.31E−03, 7.44E−03, 7.44E−03
Protein export (KEGG)	2.40E−04
Selenocompound metabolism (KEGG-InterPro)	5.37E−04
Superpathway of thiamin diphosphate biosynthesis I (PseudoCyc)	6.11E−04
Glutathione metabolism (KEGG-InterPro)	8.52E−04
Anaerobic respiration (PseudoCyc)	1.46E−03
Cationic antimicrobial peptide (CAMP) resistance (KEGG)	1.48E−03
l-Histidine biosynthesis (UniPathway)	2.81E−03
Acetyl-CoA assimilation (PseudoCyc)	4.09E−03
Beta-Alanine metabolism (KEGG)	4.21E−03
Purine metabolism (PseudoCAP, KEGG)	5.44E−03, 5.50E−03
Pantothenate and CoA biosynthesis (PseudoCAP)	6.54E−03
4-Amino-4-deoxy-alpha-l-arabinose undecaprenyl phosphate biosynthesis (UniPathway)	6.86E−03
*S*-Adenosylmethioninamine biosynthesis (UniPathway)	6.86E−03
Spermidine biosynthesis (UniPathway)	6.86E−03
Two-component regulatory systems (PseudoCAP)	6.86E−03
UDP-4-deoxy-4-formamido-beta-l-arabinose biosynthesis (UniPathway)	6.86E−03
Biosynthesis of secondary metabolites (KEGG)	7.62E−03

**p values for pathways overrepresented with up- and down-regulated genes (Fisher’s exact test implemented in the R package GeneOverlap).

^a^Pathways were downloaded from http://www.pseudomonas.com/pathways/list on 2/27/2019 and included KEGG (120), KEGG-InterPro (135), PseudoCAP (126), PseudoCyc (238), and UniPathway (165) pathways—the parentheses in this sentence are numbers of pathways per database.

The greater enrichment of down- than up-regulated genes in pathways may be due to the extensive study of log-phase cultures transforming into biofilm and the down-regulation of well-known metabolic processes. In contrast, the pathways bacteria already colonizing environments (e.g., wounds) upregulate to establish and maintain biofilm appear less well-studied and annotated.

#### Down-regulated pathways in biofilm infection (D5&D9:H2&H6)

The PAO1 pathways that became down-regulated in biofilm wound infection were initially, in the colonization stage, either on average up-regulated or unchanged vs. log-phase culture; then they typically dropped down to the expression level of the liquid culture or lower in the mature biofilm infection on D9 (Supplementary Fig. S6). One exception is the Type III Secretion System; its genes during colonization were expressed on average ~ 16-fold more vs. culture, and in biofilm infection they remained elevated above the culture level by ~ fourfold.

#### Up-regulated pathways in biofilm infection (D5&D9:H2&H6)

**Figure 5:**
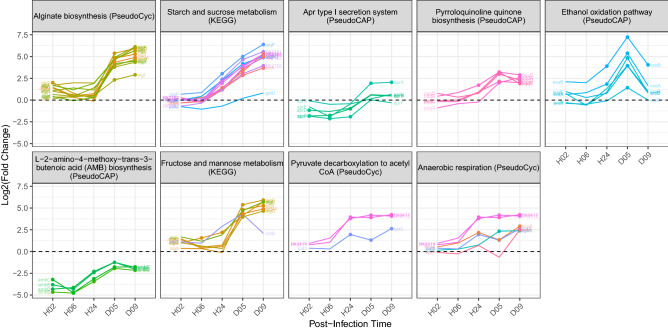
Pathways up-regulated from acute to biofilm infection. Genes of pathways over-represented with up-regulated genes (D5&D9:H2&6) are plotted vs. in-wound time (Log2FC is in-wound vs. culture). Dashed lines indicate the expression level in log-phase culture. Solid circles indicate a significant gene expression difference (FDR < 0.05) between log-phase culture and wounds at each post-infection time.

From colonization to biofilm infection, pathways enriched with up-regulated genes first changed expression little between 2 and 6 h in the wound, typically not increasing until between 6 and 24 h (Fig. 5). Additionally, these up-regulated pathways’ gene expression pattern across wound infection times is more variable than that of the down-regulated pathways. For example, the expression of the genes in the ‘Alginate biosynthesis’ and ‘Fructose and mannose metabolism’ (comprised of many of the same genes) pathways increased expression sharply from H24 to D5 and then somewhat leveled off by D9. In contrast, ‘Starch and sucrose metabolism’ genes increased almost logarithmically from H6 to D9. Other pathways’ expression was alike between the less mature (D5) and more (D9) mature biofilm infection (i.e., ‘Apr type I section system’, ‘Pyrroloquinoline quinone biosynthesis’, ‘Pyruvate decarboxylation to acetyl CoA’ and ‘AMB biosynthesis'), except the ‘Ethanol oxidation pathway’ genes rose from acute infection to day 5 of biofilm infection but then dropped on day 9.

### Less-mature vs. more-mature biofilm wound infection (D9:D5): pathway changes

Of all the comparisons between wound infection times, the smallest difference in gene expression was between Day 5 and 9. Therefore, to include more genes for analysis, albeit with a higher false discovery rate, all genes differing in expression between earlier and later wound biofilm by ≥ 1.4-fold were tested for overrepresentation into pathways. Fifty pathways were significantly overrepresented (p < 0.05) with up- or down-regulated genes as biofilm infection matured from Day 5 to 9 (Supplementary Fig. S7, Supplementary File S7).

### Colonization to acute infection (H24:H2&H6): pathway changes

The pathways up-regulated from colonization (H2&H6) to acute (H24) infection (Supplementary File S8) include pathways also up-regulated from colonization (H2&H6) to biofilm (D5&D9) infection as well as pathways uniquely up-regulated from colonization to acute infection (Supplementary Fig. S8).

### Overview of pathway changes as wound infection developed

**Figure 6:**
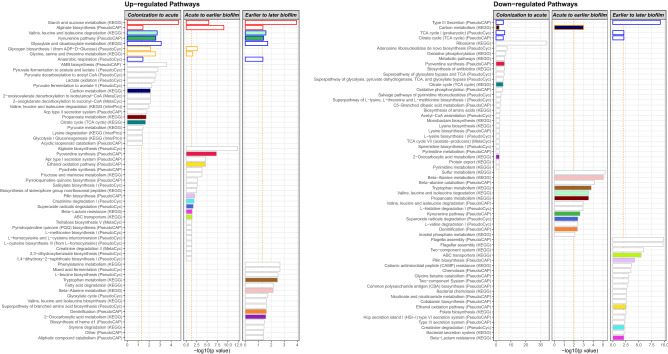
Overview of pathway changes as colonized PAO1 progressed to biofilm wound infection. Between each transition—*Colonization to acute infection* (H24:H2H6), *Acute infection to biofilm infection* (D5:H24), and *Earlier to later biofilm infection* (D9:D5)—the pathways with over-represented up- or down-regulated genes are graphed vs. the p value of the overrepresentation of the genes into the pathways; the −log10 of the p value is graphed so more significant values are larger. Vertical dashed orange lines indicate p values < 0.05 or 0.01 for the up-and down-regulated pathways, respectively (so a similar number of pathways could be graphed since more pathways were enriched with down-regulated genes). Red-outlined bars indicate pathways changed in all comparisons. Blue-outlined bars indicate pathways changed between *Colonization to acute* infection and *Earlier to later biofilm*. Orange-outlined bars indicate pathways changed between *Colonization to acute* infection and *Acute to earlier biofilm* infection. Colored bars indicate pathways changed between any two comparisons.

We graphed the p values for all the pathways that significantly changed (i.e., were overrepresented with up- or down-regulated genes) from *Colonization to acute* infection (H24:H2H6), *Acute to earlier biofilm* infection (D5:H24), and *Earlier to later biofilm* infection (D9:D5) (Fig. 6). In all three comparisons, the ‘Starch and sucrose metabolism (KEGG)’ and ‘Alginate biosynthesis (PseudoCAP)’ pathways were up-regulated, suggesting that extracellular polysaccharide was continuously or increasingly produced as the infection developed (Fig. 6, left panel, red-outlined bars).

Additionally, although the transition from *Colonization to acute* infection seems unlike the transition from *Earlier to later biofilm*, the two transitions shared several pathway changes: ‘TCA cycle’ and ‘Type III secretion’ became down-regulated, while pathways that became up-regulated included ‘Valine, leucine and isoleucine degradation (KEGG)’ (Supplementary Fig. S7), ‘Kynurenine pathway (PseudoCAP)’, ‘Glyoxylate and dicarboxylate metabolism (KEGG)’, and ‘Anaerobic respiration (PseudoCyc)’ (Fig. 6, right and left panels, blue-outlined bars).

Lower oxygen availability is suggested by the up-regulation of ‘Anaerobic respiration (PseudoCyc)’ from both *Colonization to acute* infection and *Earlier to later biofilm* infection, which is further supported by the up-regulation of ‘Pyruvate fermentation to acetate and lactate I (PseudoCyc)’ and ‘Pyruvate fermentation to acetate II (PseudoCyc)’. Furthermore, lowered oxygen from *Earlier to later biofilm* is suggested by up-regulated ‘Mixed acid fermentation (PseudoCyc)’ and ‘Denitrification (PseudoCAP)’. Denitrification is an alternative form of respiration whereby nitrate or nitrite is reduced to nitrogen gas when oxygen is limiting.

Additionally, nutrient limitation is suggested by the up-regulation of ‘Glyoxylate and dicarboxylate metabolism’ both at acute infection (H24) and mature biofilm infection (D9). The ‘Glyoxylate and dicarboxylate metabolism’ pathway allows bacteria to grow on limited carbon sources by synthesizing macromolecules (e.g., extracellular polysaccharides) from two-carbon compounds such as *ethanol* and *acetate*^15,31^. Additionally, the glyoxylate cycle is up-regulated in *P. aeruginosa* under conditions of oxidative stress^17^ and antibiotic stress, which induces oxidative stress^32^.

In acute wound infection, PAO1 may have relied on amino acids as a carbon source, as ‘Valine, leucine and isoleucine degradation (KEGG)’ (branched-chain amino acids) and ‘Glycine, serine and threonine metabolism (KEGG)’ were up-regulated. While in biofilm infection (D9), ‘Fatty acid degradation (KEGG)’ was upregulated, indicating that mature biofilm may utilize fatty acids for carbon and energy. Fatty acid utilization by *P. aeruginosa* in biofilm wound infection was previously suggested by transcriptome profiling (RNA-seq) and genome-wide insertion-mutant fitness profiling (Tn-seq): genes for long-chain fatty acid utilization were up-regulated and their mutants had attenuated growth in biofilm infection^15^.

In our study (Fig. 6), as colonizing PAO1 developed acute infection, energy utilization pathways up-regulated. Then, as acute infection progressed to biofilm, these pathways down-regulated and virulence pathways up-regulated. Finally, as biofilm infection matured, virulence pathways down-regulated, and the pathways that were up-regulated support survival despite reduced nutrients and oxygen, consistent with up-regulation of the COGs, ‘Secondary metabolites biosynthesis, transport and catabolism’, ‘Defense mechanisms’, and ‘Carbohydrate transport and metabolism’.

### Our transcriptome data vs. published *P. aeruginosa in-vitro* and *in-vivo* transcriptome data

#### Human infection vs. culture

Cornforth et al. investigated human *P. aeruginosa* infection, comparing the transcriptomes of 15 diverse human *P. aeruginosa* infections to the transcriptomes of 87 in-vitro cultures^10^. They found TCA cycle genes expressed more highly in cultures than in the human infections. Similarly, these genes in our data expressed more highly in culture than in late-stage biofilm infection, although during colonization the TCA cycle genes expressed about the same as the culture level and then dropped as biofilm infection developed (Supplementary Fig. S6).

Cornforth et al. also found core “QS regulon” genes expressed more highly in cultures, with cultures containing aggregates or biofilm expressing them most highly, than in human infections. Our data also show that expression of core QS regulon genes in log-phase culture was greater than in wounds, and as the colonizing bacteria developed into biofilm, their expression increased, but stayed below the culture level even in the most late-stage wound infection.

#### Acute pneumonia model vs. agar culture

Damron et al. analyzed the transcriptome of *P. aeruginosa* growing in lungs in an acute pneumonia murine model at 16 h post-intranasal infection vs. agar growth for 16 h^11^. Their study and ours, despite the different species (mouse vs. rabbit), different infected tissues (lung vs. skin), different culture conditions used as the denominator (agar plate culture vs. log-phase liquid culture), both found the type III secretion system vs. culture was highly expressed in vivo*.* However, in the pneumonia model, the type VI secretion system was up-regulated, but was not in the skin wounds (Supplementary Fig. S7). In both studies, heme acquisition, ferric-enterobactin transport, and pyoverdine biosynthesis genes were highly up-regulated during infection (Supplementary Fig. S4).

#### Clinical isolates planktonic vs. biofilm

Thöming et al. studied 77 *P. aeruginosa* clinical isolates (treated as replicates) that they cultured planktonically and as biofilm and identified genes expressed differentially between the two conditions^33^. They identified 103 genes that up-regulated and 40 genes that down-regulated in biofilm that they termed the core biofilm transcriptome. We correlated these genes’ planktonic-to-biofilm differential expression with our PAO1 planktonic-to-wound infection differential expression, correlating the up-regulated and down-regulated genes independently. The up-regulated genes correlated between the two datasets, significantly but weakly (H02, r = 0.22, p < 0.05; H06, r = 0.22, p < 0.05; H24, r = 0.27, p < 0.01; D5, r = 0.42, p < 0.001; D9, r = 0.29, p < 0.01). That is, the genes of the clinical isolates that up-regulated from planktonic to biofilm in vitro also tended to upregulate when log-phase PAO1 developed biofilm wound infection. Conversely and unexpectedly, the down-regulated core-biofilm genes’ expression correlated more strongly but negatively (H2, r = − 0.73, p < 0.001; H6, r = − 0.75, p < 0.001; H24, r = − 0.74, p < 0.001; D5, r = − 0.71, p < 0.001; D9, r = − 0.66, p < 0.001). That is, the 40 genes that down-regulated from planktonic to biofilm in the clinical isolates in vitro mostly up-regulated after PAO1 log-phase culture was inoculated into wounds and developed biofilm wound infection (Supplementary File S9). Twenty-two of these genes function in denitrification (*nirC, nirD, nirE, nirF, nirG, nirH, nirJ, nirL, nirM, nirN, nirQ, nirS, norB, norC, norD, norD, nosD, nosF, nosR, nosZ,* PA0526, PA2662)^34^. Thus, these denitrification genes were suppressed in in-vitro biofilm but induced in wound biofilm infection. The remaining genes function in nitrosative stress (*fhp*)^35^, arginine and proline metabolism (*aruC*, *aruG*, *aruD*, *aruB*), transport of branched-chain amino acids (*braC*), removal of superoxide radicals (*sodB*, which confers multidrug tolerance in stationary-phase bacteria)^36^, osmotic inducibility (*OsmE*) ^37^, protein folding (*groES*), dormancy (*sutA*), and virulence and antibiotic susceptibility, including to ciprofloxacin (*pfpl*)^38^.

#### Ciprofloxacin treatment

In our model, PAO1 was seeded into 3-day-old excision wounds, and after 24 h of growth, ciprofloxacin ointment was applied to the infected wounds and likely impacted gene expression. To assess this, we compared our data with that from two studies of gene expression induced by ciprofloxacin. Brazas and Hancock treated planktonic cultures (PAO1) with ciprofloxacin (1 × MIC), grew the bacteria for 2.5 h to mid-log phase, and assessed the transcriptome by microarray, identifying 492 down-regulated and 738 up-regulated genes^39^. There was no correlation between their data and ours. Murray et al. treated mid-log phase cultures (strain PA14) with ciprofloxacin at 0.5 × MIC for 30 min. and identified 8 up-regulated genes (≥ twofold change and FDR < 0.05: *recA*, *recX*, PA3413 (YebG-like), *sulA*, *lexA*, PA2288, PA1865 (Fanconi-associated nuclease 1, SAP domain), and *recN*; mean fold change ~ 6)^40^. These genes, which are largely involved in the DNA-damage response^30^, were also up-regulated by an average ~ twofold in PAO1 infecting rabbit ear wounds on Day 5, the earliest sample after ciprofloxacin treatment at Hour 24.

### When myeloid cell wound infiltration and host-wound-tissue expression of *ACOD1* for itaconate production were greatest, PAO1 itaconate catabolism genes were most up-regulated

**Figure 7:**
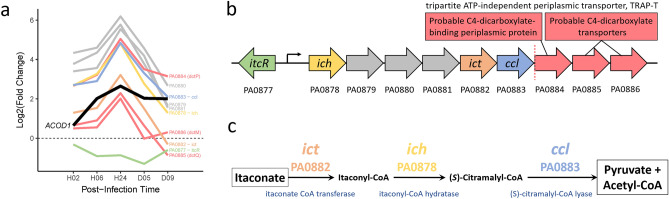
Up-regulation of itaconate catabolism genes during acute infection. (**a**) Expression of PA0877 to PA0886 (log2FC vs. culture). The horizontal dotted-black line indicates the expression of PAO1 genes in culture. The thick solid black line shows the expression of the rabbit host *ACOD1* (aka *IRG1*) gene^21^ (BioProject Accesion ID: PRJNA324375) that encodes aconitate decarboxylase 1 that catalyzes cis-aconitate to itaconate. (**b**) Schematic of the predicted operon (PA0878–PA0883) containing the three itaconate catabolism genes (*ich*, *ict*, and *ccl*) and three other genes (grey). The downstream, predicted three-gene operon shown by red arrows encodes a probable TRAP-T that may transport itaconate into PAO1. The vertical dotted red line separates the two predicted operons. (**c**) Catabolism of itaconate to pyruvate and acetyl-CoA. Panels (**b**) and (**c**) were adapted from Hanko et al.^57^.

We mapped our gene expression data onto KEGG PAO1-specific metabolic pathways^41–45^ to identify potential metabolite interactions (Supplementary Fig. S9). This revealed upregulation (4- to 32-fold) from colonization to acute infection of three PAO1 genes for catabolism of itaconate to pyruvate and acetyl-CoA (*ich*, *ict*, and *ccl*)^46^ (Fig. 7).

The up-regulation of *P. aeruginosa* itaconate catabolic genes may have been stimulated by itaconate produced by myeloid cells. Itaconate is one of the most abundant metabolites produced by myeloid cells in response to infection^47,48^. Itaconate counteracts the inflammatory effects of succinate and activates the host antioxidant response^47–49^.

Additionally, itaconate in some microbial species can inhibit isocitrate lyase, a glyoxylate cycle enzyme that enables growth on two-carbon compounds (e.g., ethanol and acetate)^50^, and can thereby be antimicrobial. By inhibiting isocitrate lyase and the glyoxylate cycle, itaconate can inhibit the intravacuolar growth of *L. pneumophila* as well as the growth of extracellular multidrug-resistant gram-positive and gram-negative bacteria^48,51–54^.

Conversely, other species such as *P. aeruginosa* (and *Yersinia pestis* by convergent evolution) can exploit itaconate as a fuel source, catabolizing it to pyruvate and acetyl-CoA^55^. The importance of itaconate as a *P. aeruginosa* fuel source in clinical infections has recently been confirmed: *P. aeruginosa* isolated from infected cystic fibrosis lungs or burn wounds carried genetic adaptations that induce macrophages to secrete greater amounts of itaconate and that enhance the pathogen’s ability to consume the metabolite as a carbon source and depend on it to produce biofilm^48^.

In our study, at 24 h of acute *P. aeruginosa* infection, myeloid cells had infiltrated the wounds to the greatest extent^21^ and the host wound tissue expressed the greatest number of transcripts of *ACOD1*, which encodes aconitate decarboxylase 1 that converts cis-aconitate into itaconate (encoded by *ACOD1*, formerly known as *IRG1*). At the same time, the *P. aeruginosa* genes for catabolizing itaconate to pyruvate and acetyl-CoA were most highly expressed (Fig. 7a). Itaconate induces the genes for its catabolism^48^. Thus, when myeloid cells were most abundant and host expression of *ACOD1* was highest, the expression of *P. aeruginosa* itaconate catabolism genes was highest, suggesting that PAO1 in acute infection was using myeloid-produced itaconate as a carbon source.

The three genes for catabolism of itaconate are on a predicted six-gene operon^56^. Upstream is the transcriptional regulator, *itcR*, that is inducible by itaconate (and mesaconate, and *cis*- and *trans*-aconitate) (Fig. 7b)^57^. [The other genes on this operon (PA0879–PA0881) are a hypothetical protein, a probable acyl-CoA dehydrogenase, and a probable ring-cleaving dioxygenase (Fig. 7b, gray arrows)]. Genes that expressed similarly to the itaconate-catabolism genes are located on the next predicted operon downstream (PA0884–PA0886; red lines in Fig. 7a and red arrows in Fig. 7b). These genes encode a probable C4-dicarboxylate transporter (a tripartite ATP-independent periplasmic transporter, TRAP-T)^58^, suggesting it transports the C4-dicarboxylate itaconate into PAO1.

## Conclusions

*Pseudomonas aeruginosa* strain PAO1 from log-phase culture was inoculated into 3-day-old full-thickness excision wounds. The pathogen first colonized, then acutely infected, and finally biofilm-infected the wounds. At 24 h post-inoculation, the bacteria had increased by ~ 2 logs, coinciding with the peak of infiltration of neutrophils and macrophages into the wounds, as we previously reported for this experiment^21^. Additionally at 24 h post-inoculation, the acutely infected wounds were treated with topical ciprofloxacin (another antibiotic may have affected the transcriptome differently^10^) and antimicrobial dressing, and by Days 5 and 9 biofilm infection had developed^21^. As infection developed in this model, the milieu changed, owing to the immune response, bacterial activities, and healing (epithelial gap was ~ 30% smaller by D9)^21^.

After PAO1 had colonized the wound for 2 h, COGs related to the liquid culture’s elevated metabolism and cell replication were mostly down-regulated. Simultaneously, the up-regulated COGs included ‘Defense mechanisms’, ‘Inorganic ion transport and metabolism’, ‘Intracellular trafficking, secretion, and vesicular transport’, and ‘Cell motility’—reflecting wound adaptation.

Between colonization and the development of biofilm infection, more genes were up-regulated than were down-regulated. However, the down-regulated genes were overrepresented in more pathways, possibly because of the extensive historical study of liquid-cultured bacteria transforming into biofilm that is associated with down-regulation of well-known annotated pathways of metabolism. Conversely, pathways up-regulated by bacteria already colonizing wounds to establish chronic biofilm infection are apparently less well known and annotated.

From colonization to mature biofilm infection, pathways required for extracellular polysaccharide production, ‘Starch and sucrose metabolism’ and ‘Alginate biosynthesis’, were increasingly up-regulated. Coincidently, from *Colonization to acute* infection and from *Earlier to later biofilm* infection, oxygen availability evidently decreased, suggested by the up-regulation of anaerobic respiration and fermentation pathways that produce *ethanol,* a potential carbon source for feeding polysaccharide production.

Available carbon sources apparently shifted as infection developed. The pathway of ‘Valine, leucine and isoleucine degradation’ (i.e., branched chain amino acids, BCAAs) was up-regulated from *Colonization to acute* infection as well as *Earlier to later biofilm*. *Earlier to later biofilm* was additionally associated with up-regulation of ‘Fatty acid degradation’. BCAA and fatty acid degradation produce *acetyl-CoA*^59,60^*,* potentially feeding polysaccharide production.

Itaconate appears to have been an additional carbon source used by PAO1 during acute infection. Itaconate is one of the most highly produced metabolites by myeloid cells in response to infection. When myeloid cells had infiltrated the wound to the greatest extent (reported in our analysis of the wound tissue response to this PAO1 infection^21^) and the wound tissue was most highly expressing the itaconate-production gene *ACOD1* (aka *IRG1*)^21^, PAO1 was most highly expressing itaconate catabolism genes. Itaconate catabolism produces pyruvate and *acetyl-CoA*. Catabolism of itaconate has been suggested as a potential drug target for inducing cell death by carbohydrate limitation^61^.

When the 2-carbon compounds *ethanol* and *acetate*—such as produced under low oxygen and as end products of BCAA, fatty acid, and itaconate catabolism—are available and glucose is not, the pathway of ‘Glyoxylate and dicarboxylate metabolism’ bypasses the TCA cycle to permit bacterial growth^50^ by feeding 2-carbon compounds into biomass^54^. The ‘Glyoxylate and dicarboxylate metabolism’ pathway was up-regulated from *Colonization to acute* infection and *Earlier to later biofilm*, supporting its importance for *P. aeruginosa* to synthesize macromolecules in oxygen- and nutrient-limited wounds.

In addition to the role of the glyoxylate shunt (isocitrate lyase, *aceA*; and malate synthase, *glcB*) in carbon metabolism, it has previously been demonstrated in *P. aeruginosa* to be up-regulated under conditions of oxidative stress^17^, antibiotic stress (induces oxidative stress^32^), and host infection^15^; moreover, the glyoxylate shunt, particularly *aceA* is required for pathogenesis in *P. aeruginosa* and other pathogenic bacterial^17–20^. Also, metabolic flux through the glyoxylate shunt is increased in *E. coli* experiencing superoxide stress^62^. In our study, the glyoxylate shunt genes, *aceA* and *glcB,* and the ‘Glyoxylate and dicarboxylate metabolism’ pathway increased expression from colonization to active infection, which may have been stimulated, at least in part, by neutrophil oxidative burst, because at 24 h post-inoculation of wounds, during active wound infection, wound infiltration with neutrophils was greatest^21^. The potential clinical relevance of the glyoxylate shunt in managing antibiotic and oxidative stress is suggested by its upregulation in some clinical isolates from urinary tract infections and CF lung^63–65^. Thus, the glyoxylate shunt may warrant further investigation as a drug target^54,66^. It is absent in animals, and its inhibition in some bacterial species (not *P. aeruginosa*) by itaconate, paradoxically, can be antimicrobial^67^.

Our study for the first time analyzed the *P. aeruginosa* transcriptome from wound colonization to biofilm wound infection and revealed pathways the pathogen evidently uses to colonize wounds and develop acute and biofilm infection.

## Methods

Details of the study regarding characterization of the wound morphology and wound tissue transcriptome are described in Karna et al.^21^. The study is reported in accordance with ARRIVE guidelines**.**

### Animal protocol

Adult female New Zealand white rabbits (3–5 kg) from Charles River Laboratories International, Inc. were acclimated to standard housing, fed ad libitum, and kept in individual cages under constant temperature (22 °C) and humidity with a 12-h light dark cycle. Research was conducted in compliance with the Animal Welfare Act, the implementing Animal Welfare regulations, and the principles of the Guide for the Care and Use of Laboratory Animals, National Research Council. The Institutional Animal Care and Use Committee at the US Army Institute of Surgical Research (USAISR, Fort Sam Houston, TX) approved all research conducted in this study. The facility where this research was conducted is fully accredited by the AAALAC. The animal model was as previously described^21^ and is briefly summarized in Fig. 1. A total of 89 animals were used for this study, which includes two biological repeats at Hrs 2, 6, 24 and Day 9, and three repeats at Day 5. All animals were included in the study; however, only two of the three Day-5 biological repeats with the highest CFU counts was analyzed^21^. All experiments were performed in accordance with relevant guidelines and regulations. Prior to the surgery for creating full-thickness dermal wounds, rabbits were anesthetized by intramuscular injection of a mixture of ketamine (22.5 mg/kg) and xylazine (3.5 mg/kg). The inner surface of the ear was injected intradermally with 1% lidocaine with epinephrine before making six, 6-mm-diameter full-thickness dermal wounds down to the perichondrium on the ventral surface, which were then dressed with Tegaderm. Animals were euthanized by an intravenous injection of a euthanasia solution (Fatal-Plus TM).

### *Pseudomonas aeruginosa* growth and seeding into wounds

Seeding of *P. aeruginosa* was as previously described^21^. Briefly, PAO1 (University of Washington subline) was grown on blood agar plates overnight at 37 °C before being subcultured into 10 mL of tryptic soy broth (TSB) and grown at 37 °C until log-phase (~ 0.1 OD_600_). The bacteria were then washed with PBS, centrifuged (4000 rpm for 10 min at 20 °C), and resuspended in PBS to ~ 0.2 OD_600_ equal to 10^6^ CFU/10 μl. Ten μl was then seeded into 6-mm-diameter full-thickness dermal wounds on the rabbit ear ventral surface.

### Total RNA extraction, quantitative RT-PCR, and RNA sequencing

The methods were previously described for the analysis of the wound tissue transcriptome^21^. For the analysis of the *P. aeruginosa* transcriptome described herein, the dorsal skin of the ear was removed prior to the wound harvest using an 8-mm punch. The harvested tissue was immediately frozen in liquid nitrogen. The RNA was isolated from seven wounds (to include the wound edges) from each animal. The remaining five wounds were used to determine viable counts, scanning electron microscopy, epithelial gap, and immunohistochemistry as previously reported^21^. To obtain total RNA, the frozen tissues were homogenized in TRIzol (Life Technologies) using the T25 ULTRATURRAX at 20,000 rpm (IKA, Germany). The RNA was purified using an RNEasy Mini-Kit (Qiagen, MD). Genomic DNA was removed by treatment with DNAse I (Ambion), and the absence of DNA contamination was confirmed using a minus-reverse transcriptase control demonstrating a Ct value 10 cycles higher than the reverse transcribed samples. The purified RNA was reverse transcribed to cDNA using the iScript Select cDNA synthesis kit (Bio-Rad). The RNA was quantified (A260/A280, NanoDrop, Thermo Scientific, Waltham, MA) and checked for purity (2200 TapeStation, Agilent Technologies, Cedar Creek, TX). The RNA from each wound was individually isolated and the purity was checked before pooling. Each RNA sequence determination was from RNA pooled from 28 to 30 infected wounds or 18–21 non-infected wounds (a feasible sample size for this exploratory research). Total RNA was submitted to Otogenetics Corporation (Norcross, GA, USA) for synthesis of illumina libraries. Briefly, the integrity and purity of total RNA were assessed by OD260/280 using the Agilent Bioanalyzer. Total RNA (5 µg) was depleted of rRNA using the RiboZero Magnetic Gold Kit Epidemiology kit (Epicentre). cDNA was generated from the depleted RNA using TruSeq Stranded Total RNA Sample Preparation kit (Illumina). The resulting cDNA was purified, fragmented by sonication (BioRuptor, Diagenode, Inc.), and profiled using the Agilent Bioanalyzer. Illumina libraries were made from the qualified, fragmented cDNA using the SPRIworks HT Reagent Kit (Beckman Coulter, Inc.) on the Biomek FXp. The quality, quantity, and size distribution of the Illumina libraries were determined using an Agilent Bioanalyzer 2100 or Tapestation. The libraries were then submitted to US Army Center for Environmental Health Research (USACEHR, Fort Detrick, MD, USA) and Forsyth Institute (Cambridge, MA, USA) for Nextseq 500 sequencing. Single-end 75 bp nucleotide reads were generated and checked for data quality using FASTQC (Babraham Institute, Cambridge, UK).

### Bioinformatic analyses

Illumina generated RNA-Seq reads were mapped to the genomic sequence of *P. aeruginosa* PAO1 (Genbank Accession NC_002516.2) using Bowtie2 version 2.1.0^68^. The mapped reads were separated to strand-specific forward and reverse-complement profiles (Samtools, Tools for alignments in the SAM format version 1.2^69^), which were used to calculate read counts for the 5572 GenBank-annotated *P. aeruginosa* protein coding genes (Supplementary File S10). One of the biological repeats at the 6-h time point was removed from the analysis because of low read counts. Differential gene expression was calculated from the gene counts using DESeq2 Bioconductor package version 1.10.1^70^ under the R environment version 3.2.2^71^. For principal component analysis, counts were normalized (DESeq2 rlog function) and the R function prcomp (scaled = True) was used to calculate the principal components. COGs were obtained from pseudomonas.com/cog/list for PAO1 based on the 2014 analysis available at NCBI's COG database^29^. *P. aeruginosa* PAO1 (Reference) Pathway Annotations were obtained from pseudomonas.com/pathways/list. Statistical analysis was performed using the R software environment for statistical computing and graphics^71^. False discovery rate of 0.05 was used unless otherwise indicated. Overrepresentation of genes in COGs and pathways was calculated using Fisher's exact test, p < 0.05, implemented in R package GeneOverlap version 1.10.0^72^.

## Supplementary Information

Dataset S1.

Dataset S2.

Dataset S3.

Dataset S4.

Dataset S5.

Dataset S6.

Dataset S7.

Dataset S8.

Dataset S9.

Dataset S10.

Supplementary Figures.

## Data Availability

The RNA-seq sequence data used in this study were deposited to the NCBI Sequence Read Archive (SRA) database and are available through the BioProject Accession ID: PRJNA700644.
