# Supplementary material for: Pseudomonas aeruginosa transcriptome adaptations from colonization to biofilm infection of skin wounds
Source: Sci Rep. 2021 Oct 19;11:20632. doi: 10.1038/s41598-021-00073-4 (PMC8526614; doi:10.1038/s41598-021-00073-4)
Supplement: Supplementary file 11 — Supplementary Figures. [file 41598_2021_73_MOESM11_ESM.pdf]

Pseudomonas aeruginosa transcriptome adaptations from colonization to biofilm infection of skin wounds

Peter D'Arpa, S. L. Rajasekhar Karna, Tsute Chen, Kai P. Leung

View using Continuous Scroll (Preview) or Enable Scrolling (Acrobat).

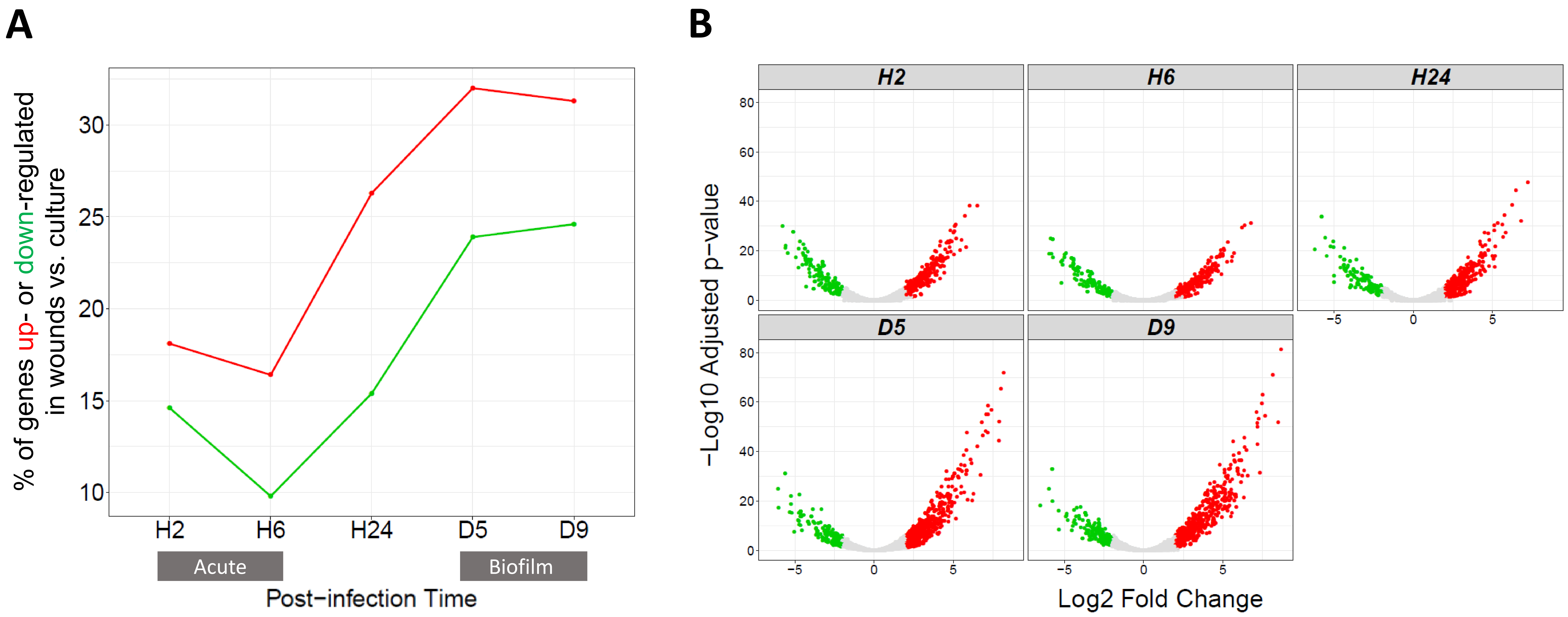

A
B
% of genes up- or down-regulated in wounds vs. culture
30
25
20
15
10
H2
H6
H24
D5
D9
Acute
Biofilm
Post−infection Time
H2
H6
H24
D5
D9
−Log10 Adjusted p−value
80
60
40
20
0
−5
0
5
Log2 Fold Change

S1 Fig. Global gene expression of PAO1 in wounds vs. log-phase culture. **(A)** The percentage of genes with up-(red) and down(green)-regulated expression in wounds (false discovery rate, FDR < 0.05). **(B)** Volcano plots.

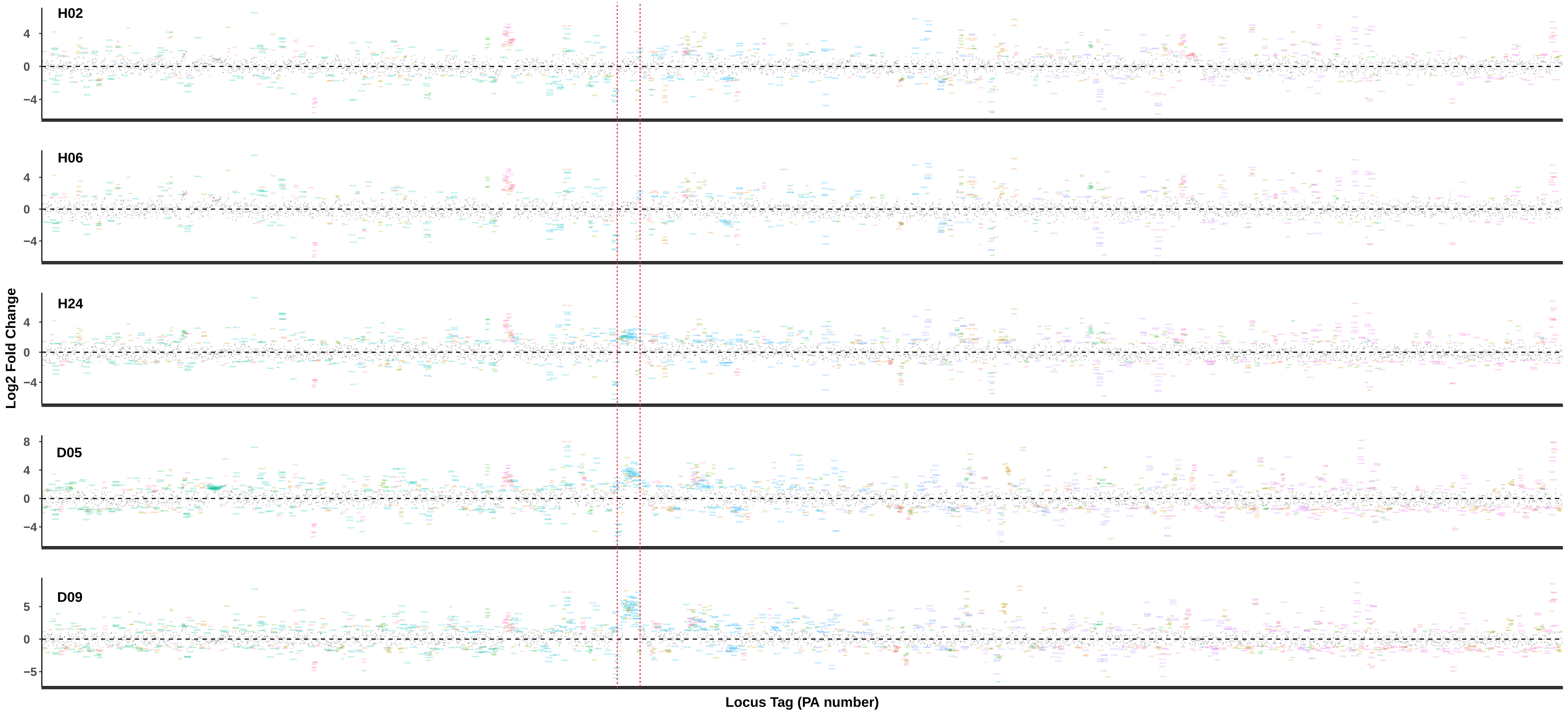
H02
H06
H24
D05
D09
4
0
−4
8
5
−5
Log2 Fold Change
Locus Tag (PA number)

S2 Fig. Log2 Fold Change of PAO1 genes at in-wound times vs. log-phase culture. Genes are ordered by their position in the genome. The red dotted lines delineate PA2134 to PA2193. Gene color is alphabetical.

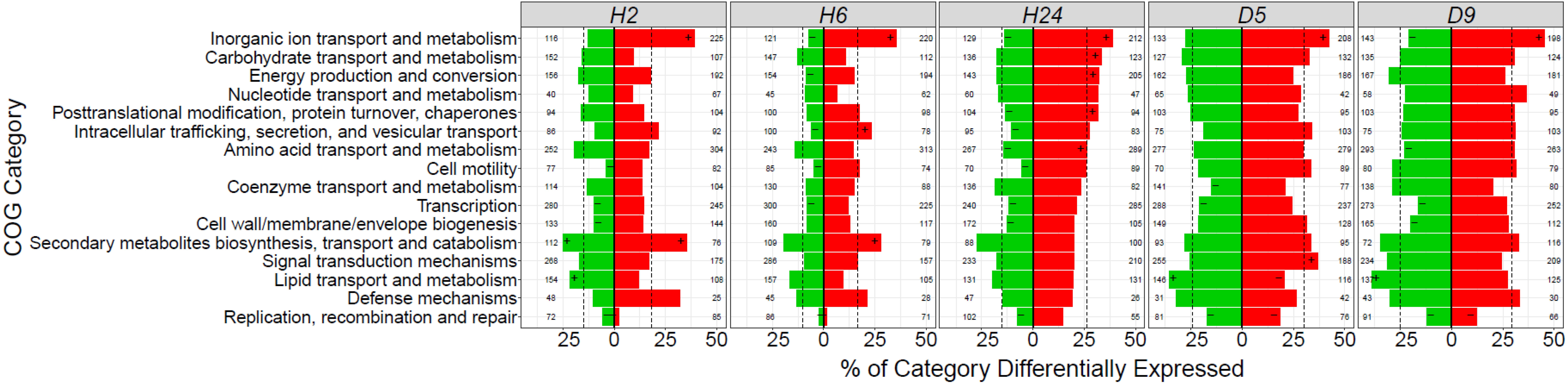

H2
H6
H24
D5
D9
Inorganic ion transport and metabolism
Carbohydrate transport and metabolism
Energy production and conversion
Nucleotide transport and metabolism
Posttranslational modification, protein turnover, chaperones
Intracellular trafficking, secretion, and vesicular transport
Amino acid transport and metabolism
Cell motility
Coenzyme transport and metabolism
Transcription
Cell wall/membrane/envelope biogenesis
Secondary metabolites biosynthesis, transport and catabolism
Signal transduction mechanisms
Lipid transport and metabolism
Defense mechanisms
Replication, recombination and repair
COG Category
% of Category Differentially Expressed
25
0
25
50

S3 Fig. Percent of genes in COG categories differentially expressed at each in-wound time (vs. log-phase culture). COG categories significantly overrepresented (+) or underrepresented (-) relative to the proportion of all significant up-regulated or down-regulated genes (Fisher's exact test, $p<0.05$, implemented in R package GeneOverlap version 1.10.0). Dotted lines indicate the percent down- and up-regulated genes averaged across all COG categories. The COG categories are arranged from top to bottom in descending order of the percent of significantly up-regulated genes at 24 hrs. The numbers in the panels are the counts of genes per COG category (*Pseudomonas Genome DB*, PAO1 COG mappings) expressed less or more so than in log-phase culture (regardless of fold-change or FDR).

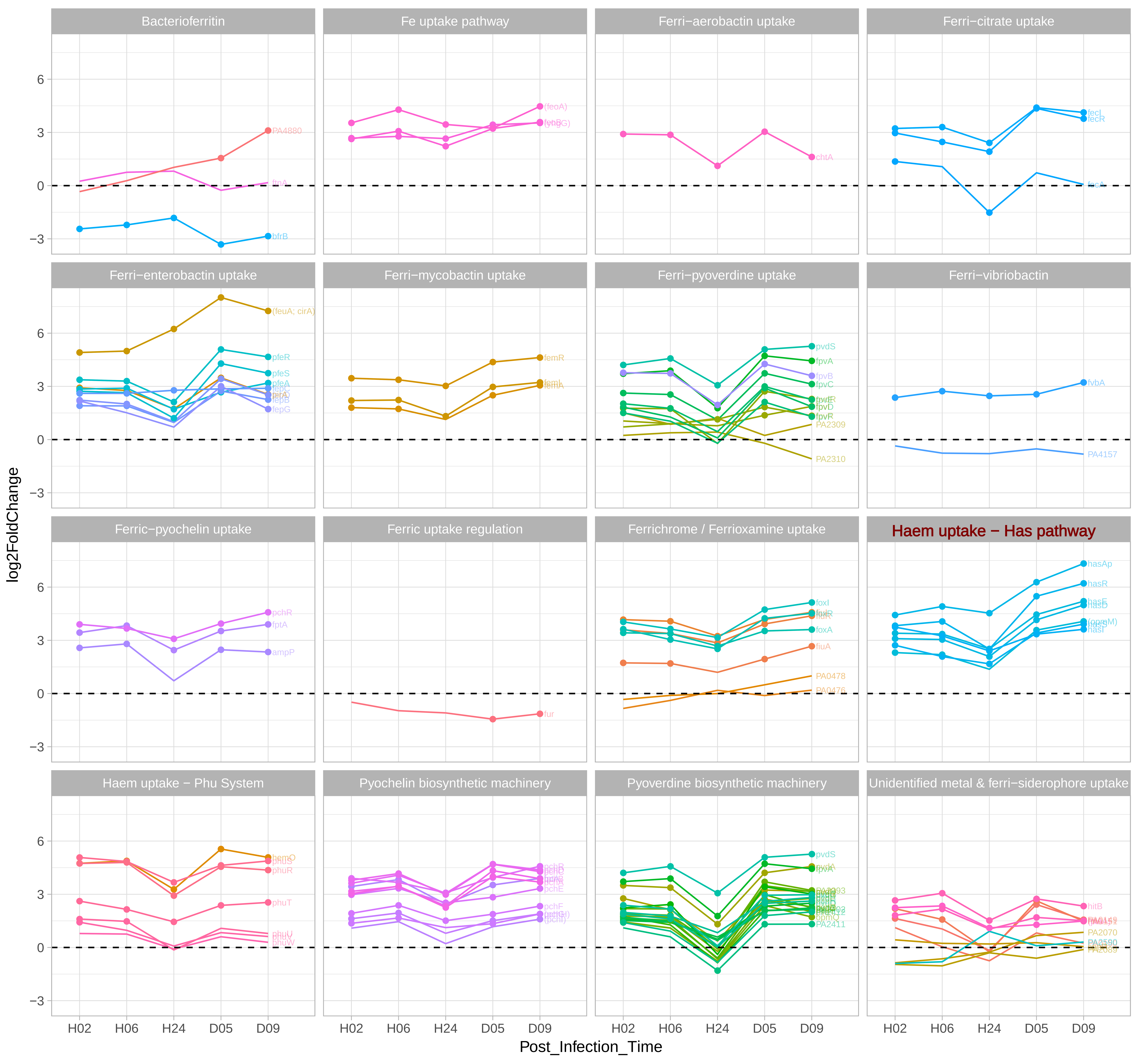

Bacterioferritin
Fe uptake pathway
Ferri−aerobactin uptake
Ferri−citrate uptake
Ferri−enterobactin uptake
Ferri−mycobactin uptake
Ferri−pyoverdine uptake
Ferri−vibriobactin
Ferric−pyochelin uptake
Ferric uptake regulation
Ferrichrome / Ferrioxamine uptake
Haem uptake − Has pathway
Haem uptake − Phu System
Pyochelin biosynthetic machinery
Pyoverdine biosynthetic machinery
Unidentified metal & ferri−siderophore uptake
PA4880
bfrB
(feoA)
chtA
fecI
fecR
(feuA; cirA)
pfeR
pfeS
pfeA
fepG
femR
femA
pvdS
fpvA
fpvB
fpvC
fpvD
fpvR
PA2309
PA2310
fvbA
PA4157
pchR
fptA
ampP
fur
foxI
foxA
fiuA
PA0478
PA0476
hasAp
hasR
hasI
hemO
phuS
phuR
phuT
pchF
pchE
(pchI)
PA2411
hitB
PA2070
log2FoldChange
6
3
0
−3
H02
H06
H24
D05
D09
Post_Infection_Time

S4 Fig. Expression of COG, 'Inorganic ion transport and metabolism', genes in wound infection vs. log-phase culture. A solid circle indicates a significant difference in the expression of the gene in the wound vs. culture.

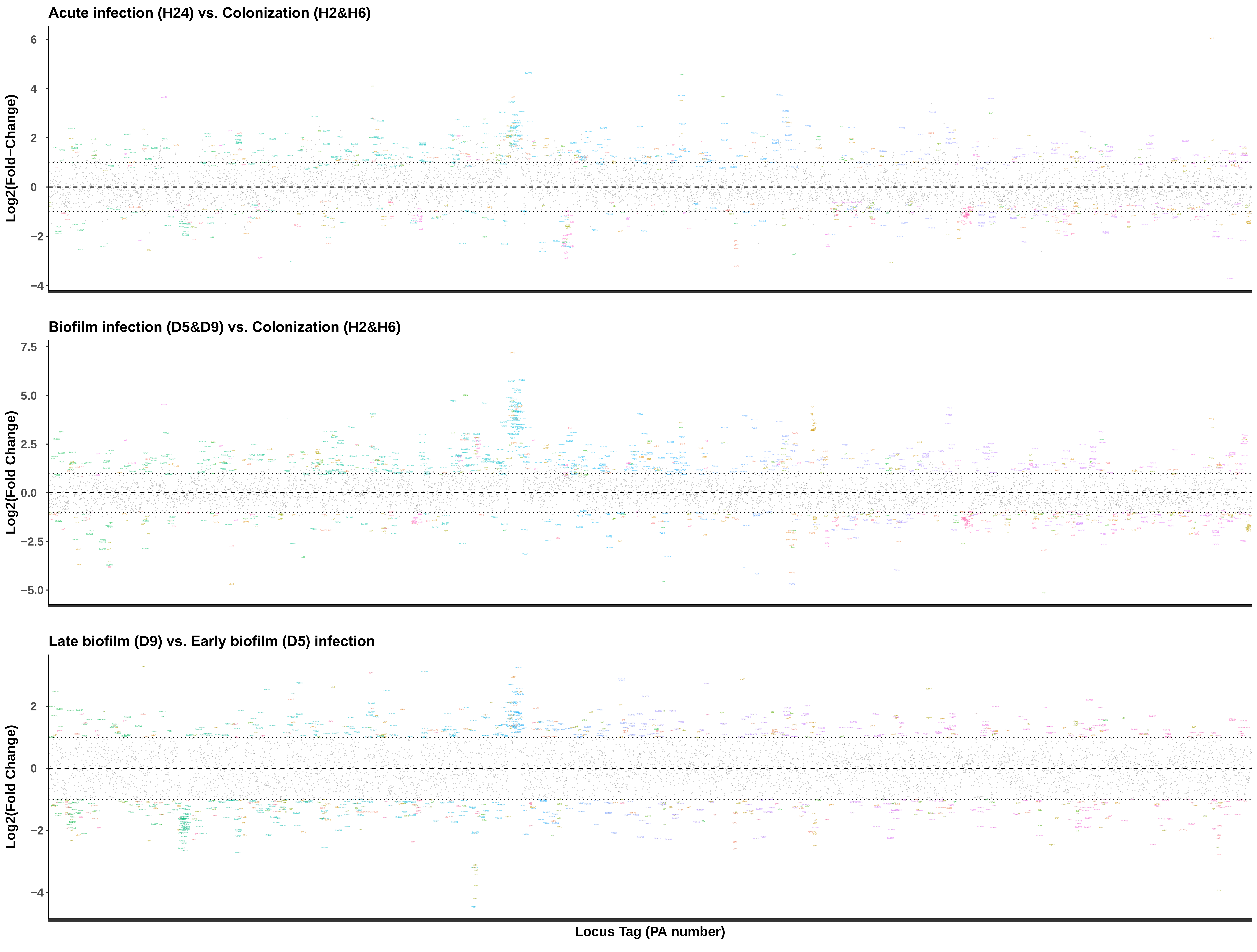
Acute infection (H24) vs. Colonization (H2&H6)
Log2(Fold−Change)
6
4
2
0
−2
−4
Biofilm infection (D5&D9) vs. Colonization (H2&H6)
Log2(Fold Change)
7.5
5.0
2.5
0.0
−2.5
−5.0
Late biofilm (D9) vs. Early biofilm (D5) infection
Log2(Fold Change)
2
0
−2
−4
Locus Tag (PA number)

S5 Fig. Changes in PAO1 gene expression as wound infection developed: colonization to acute infection, colonization to biofilm infection, and earlier to later biofilm infection.

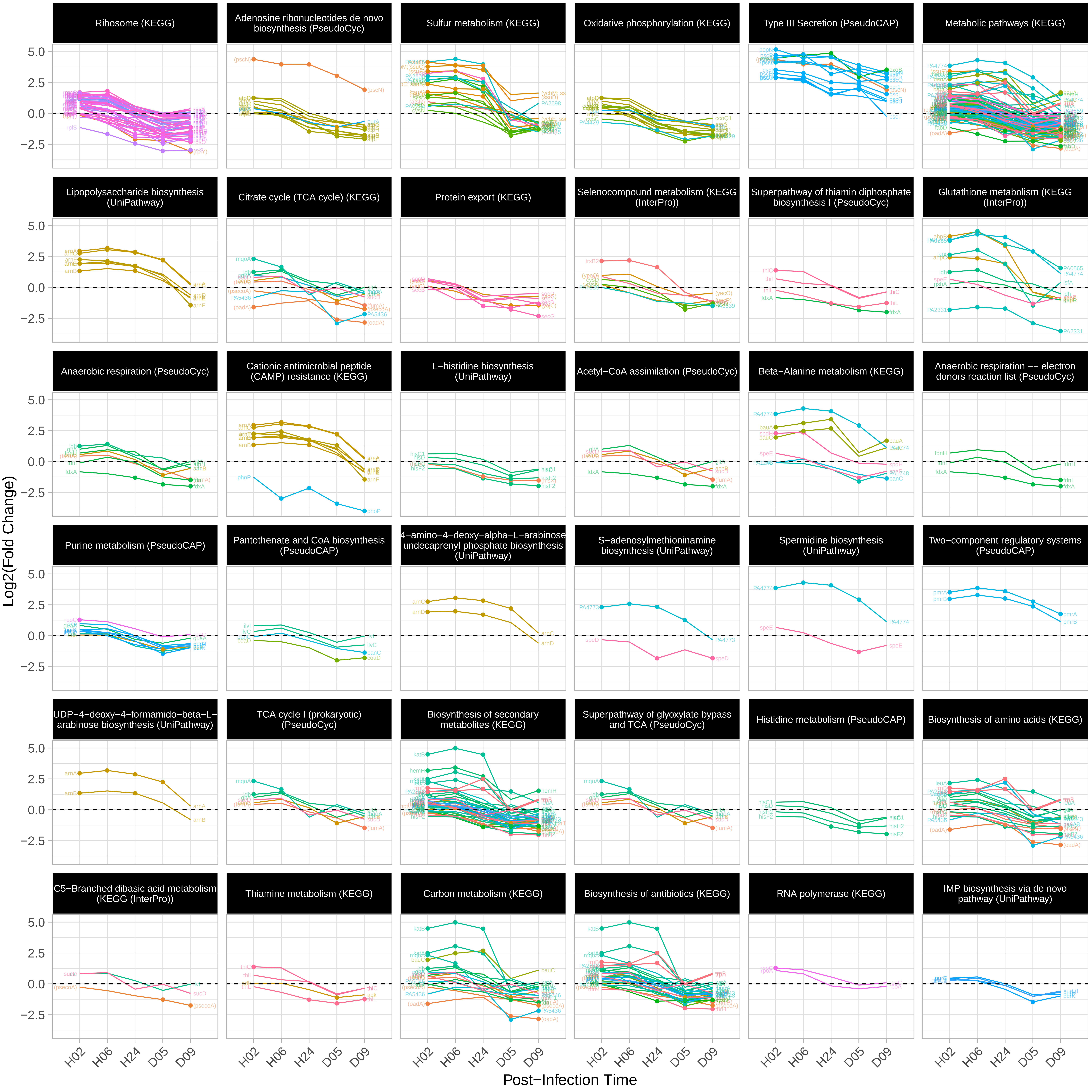
Ribosome (KEGG)
Adenosine ribonucleotides de novo biosynthesis (PseudoCyc)
Sulfur metabolism (KEGG)
Oxidative phosphorylation (KEGG)
Type III Secretion (PseudoCAP)
Metabolic pathways (KEGG)
Lipopolysaccharide biosynthesis (UniPathway)
Citrate cycle (TCA cycle) (KEGG)
Protein export (KEGG)
Selenocompound metabolism (KEGG (InterPro))
Superpathway of thiamin diphosphate biosynthesis I (PseudoCyc)
Glutathione metabolism (KEGG (InterPro))
Anaerobic respiration (PseudoCyc)
Cationic antimicrobial peptide (CAMP) resistance (KEGG)
L−histidine biosynthesis (UniPathway)
Acetyl−CoA assimilation (PseudoCyc)
Beta−Alanine metabolism (KEGG)
Anaerobic respiration −− electron donors reaction list (PseudoCyc)
Purine metabolism (PseudoCAP)
Pantothenate and CoA biosynthesis (PseudoCAP)
4−amino−4−deoxy−alpha−L−arabinose undecaprenyl phosphate biosynthesis (UniPathway)
S−adenosylmethioninamine biosynthesis (UniPathway)
Spermidine biosynthesis (UniPathway)
Two−component regulatory systems (PseudoCAP)
UDP−4−deoxy−4−formamido−beta−L−arabinose biosynthesis (UniPathway)
TCA cycle I (prokaryotic) (PseudoCyc)
Biosynthesis of secondary metabolites (KEGG)
Superpathway of glyoxylate bypass and TCA (PseudoCyc)
Histidine metabolism (PseudoCAP)
Biosynthesis of amino acids (KEGG)
C5−Branched dibasic acid metabolism (KEGG (InterPro))
Thiamine metabolism (KEGG)
Carbon metabolism (KEGG)
Biosynthesis of antibiotics (KEGG)
RNA polymerase (KEGG)
IMP biosynthesis via de novo pathway (UniPathway)
Log2(Fold Change)
5.0
2.5
0.0
−2.5
H02
H06
H24
D05
D09
Post−Infection Time

S6 Fig. Pathways down-regulated from acute to biofilm infection. Pathways with over-represented down-regulated genes in biofilm infection (D5&D9:H2&6) are shown with their genes plotted vs. in-wound time. Dashed lines indicate the expression level in log-phase culture. Solid circles indicate a significant gene expression difference (FDR < 0.05) between log-phase culture and wounds at each in-wound time.

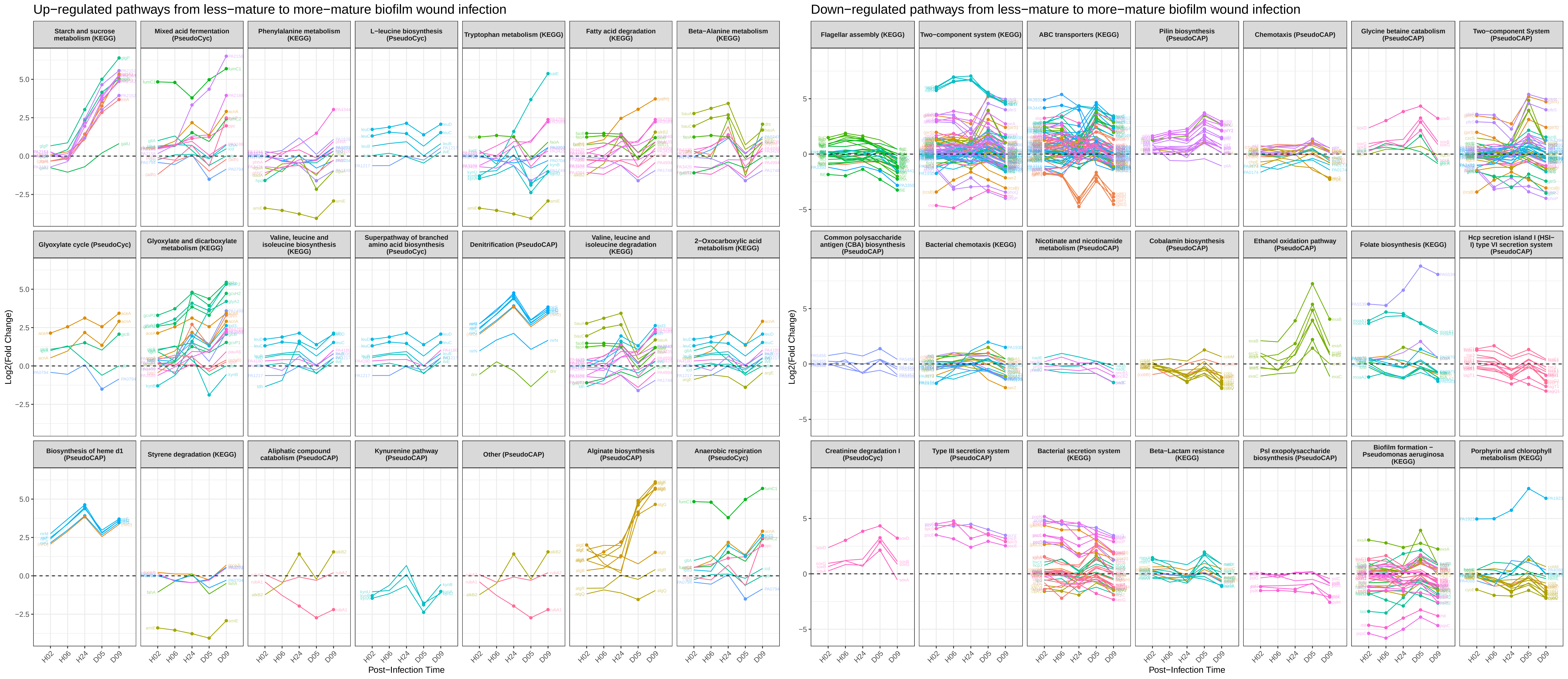
Up−regulated pathways from less−mature to more−mature biofilm wound infection
Starch and sucrose metabolism (KEGG)
Mixed acid fermentation (PseudoCyc)
Phenylalanine metabolism (KEGG)
L−leucine biosynthesis (PseudoCyc)
Tryptophan metabolism (KEGG)
Fatty acid degradation (KEGG)
Beta−Alanine metabolism (KEGG)
Glyoxylate cycle (PseudoCyc)
Glyoxylate and dicarboxylate metabolism (KEGG)
Valine, leucine and isoleucine biosynthesis (KEGG)
Superpathway of branched amino acid biosynthesis (PseudoCyc)
Denitrification (PseudoCAP)
Valine, leucine and isoleucine degradation (KEGG)
2−Oxocarboxylic acid metabolism (KEGG)
Biosynthesis of heme d1 (PseudoCAP)
Styrene degradation (KEGG)
Aliphatic compound catabolism (PseudoCAP)
Kynurenine pathway (PseudoCAP)
Other (PseudoCAP)
Alginate biosynthesis (PseudoCAP)
Anaerobic respiration (PseudoCyc)
Log2(Fold Change)
5.0
2.5
0.0
−2.5
H02
H06
H24
D05
D09
Post−Infection Time
Down−regulated pathways from less−mature to more−mature biofilm wound infection
Flagellar assembly (KEGG)
Two−component system (KEGG)
ABC transporters (KEGG)
Pilin biosynthesis (PseudoCAP)
Chemotaxis (PseudoCAP)
Glycine betaine catabolism (PseudoCAP)
Two−component System (PseudoCAP)
Common polysaccharide antigen (CBA) biosynthesis (PseudoCAP)
Bacterial chemotaxis (KEGG)
Nicotinate and nicotinamide metabolism (PseudoCAP)
Cobalamin biosynthesis (PseudoCAP)
Ethanol oxidation pathway (PseudoCAP)
Folate biosynthesis (KEGG)
Hcp secretion island I (HSI−I) type VI secretion system (PseudoCAP)
Creatinine degradation I (PseudoCyc)
Type III secretion system (PseudoCAP)
Bacterial secretion system (KEGG)
Beta−Lactam resistance (KEGG)
Psl exopolysaccharide biosynthesis (PseudoCAP)
Biofilm formation − Pseudomonas aeruginosa (KEGG)
Porphyrin and chlorophyll metabolism (KEGG)
Log2(Fold Change)
5
0
−5
H02
H06
H24
D05
D09
Post−Infection Time

S7 Fig. Pathways up- and down-regulated from less-mature (D5) to more-mature (D9) *P. aeruginosa* wound infection. Genes in the pathways are plotted vs. post-infection time. Dashed lines indicate the expression level in log-phase culture. Solid circles indicate a significant gene expression difference (FDR < 0.05) between log-phase culture and wounds at each post-infection time.

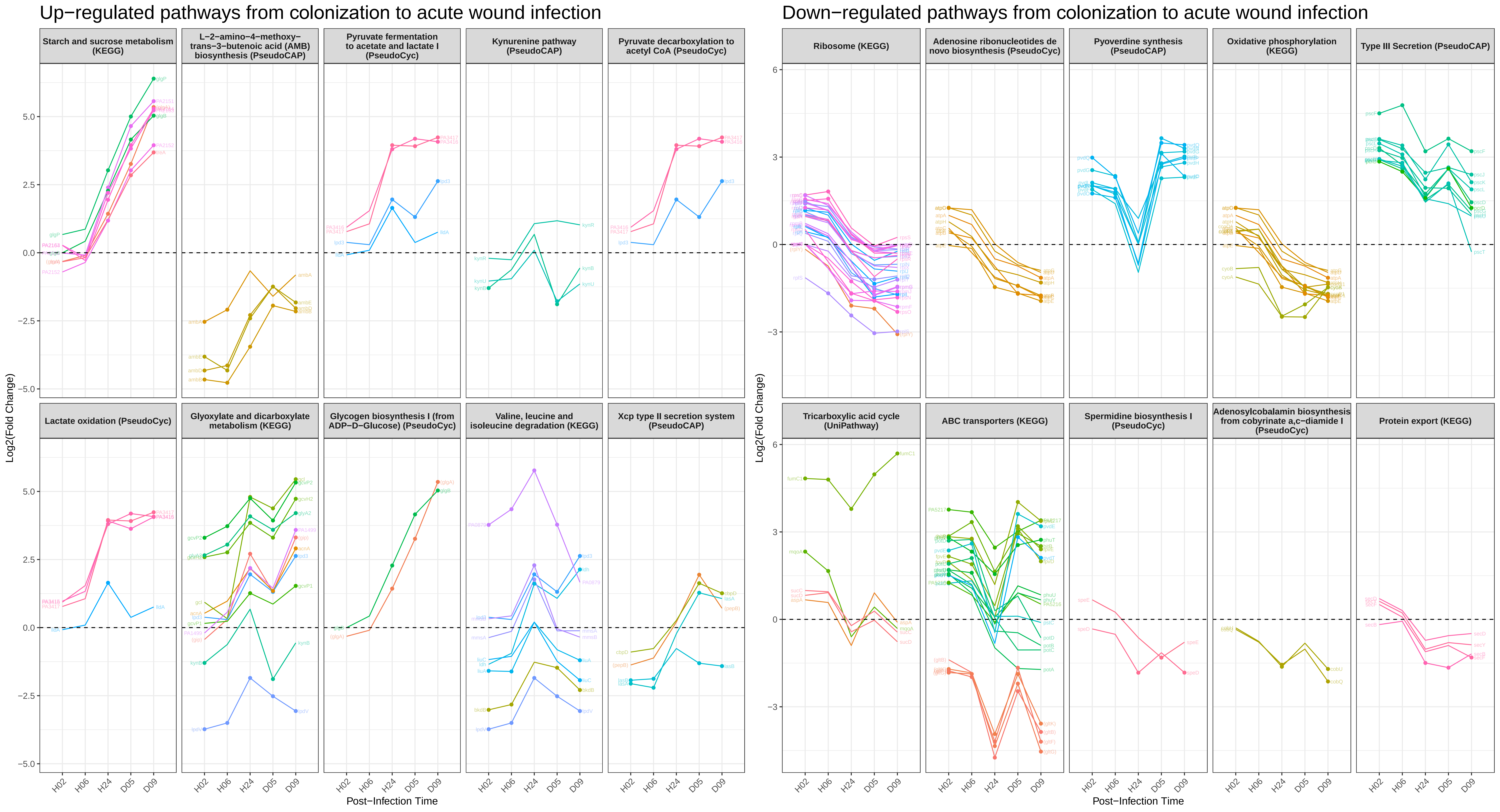

Up−regulated pathways from colonization to acute wound infection
Down−regulated pathways from colonization to acute wound infection
Starch and sucrose metabolism (KEGG)
L−2−amino−4−methoxy−trans−3−butenoic acid (AMB) biosynthesis (PseudoCAP)
Pyruvate fermentation to acetate and lactate I (PseudoCyc)
Kynurenine pathway (PseudoCAP)
Pyruvate decarboxylation to acetyl CoA (PseudoCyc)
Lactate oxidation (PseudoCyc)
Glyoxylate and dicarboxylate metabolism (KEGG)
Glycogen biosynthesis I (from ADP−D−Glucose) (PseudoCyc)
Valine, leucine and isoleucine degradation (KEGG)
Xcp type II secretion system (PseudoCAP)
Ribosome (KEGG)
Adenosine ribonucleotides de novo biosynthesis (PseudoCyc)
Pyoverdine synthesis (PseudoCAP)
Oxidative phosphorylation (KEGG)
Type III Secretion (PseudoCAP)
Tricarboxylic acid cycle (UniPathway)
ABC transporters (KEGG)
Spermidine biosynthesis I (PseudoCyc)
Adenosylcobalamin biosynthesis from cobyrinate a,c−diamide I (PseudoCyc)
Protein export (KEGG)
Log2(Fold Change)
5.0
2.5
0.0
−2.5
−5.0
6
3
0
−3
H02
H06
H24
D05
D09
Post−Infection Time

S8 Fig. Pathways up- and down-regulated from colonization (H2&H6) to acute (H24) infection. Pathways with over-represented up- or down-regulated genes in biofilm infection (H24:H2&6) are shown with their genes plotted vs. time after infection. Dashed lines indicate the expression level in log-phase culture. Solid circles indicate a significant gene expression difference (FDR < 0.05) between log-phase culture and wounds at each post-infection time.

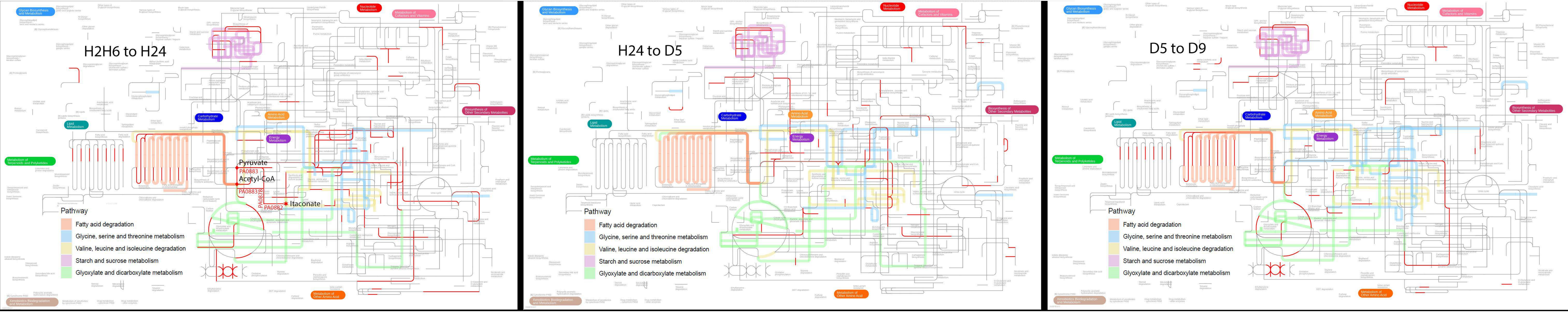

H2H6 to H24
Pyruvate
PA0883
Acetyl-CoA
PA0878
Itaconate
PA0882
Pathway
Fatty acid degradation
Glycine, serine and threonine metabolism
Valine, leucine and isoleucine degradation
Starch and sucrose metabolism
Glyoxylate and dicarboxylate metabolism
H24 to D5
Pathway
Fatty acid degradation
Glycine, serine and threonine metabolism
Valine, leucine and isoleucine degradation
Starch and sucrose metabolism
Glyoxylate and dicarboxylate metabolism
D5 to D9
Pathway
Fatty acid degradation
Glycine, serine and threonine metabolism
Valine, leucine and isoleucine degradation
Starch and sucrose metabolism
Glyoxylate and dicarboxylate metabolism

S9 Fig. Mapping of up-regulated genes onto KEGG pathways specific for *P. aeruginosa* strain PAO1. Up-regulated genes, about 450 for each comparison (selected by adjusting the FDR cutoff), were mapped (red lines) onto *P. aeruginosa* PAO1 metabolic pathways (pae01100) using Interactive Pathways Explorer v3 (42).
